# Efficacy and Safety of a Botanical Formula Fuzheng Huayu for Hepatic Fibrosis in Patients with CHC: Results of a Phase 2 Clinical Trial

**DOI:** 10.1155/2022/4494099

**Published:** 2022-07-15

**Authors:** Tarek Hassanein, Dean Tai, Chenghai Liu, Terry D. Box, Myron J. Tong, Lorenzo Rossaro, Renee Pozza, Jeffrey S. Glenn, Ramsey Cheung, Ammar Hemaidan, Yingchun He, Cynthia Behling, Xiqi Hu, Hala Makhlouf, Haina Fan, Yayun Ren, Elaine Lay Khim Chng, Ping Liu, John M. Vierling

**Affiliations:** ^1^SCTI Research Foundation, Coronado, CA, USA; ^2^HistoIndex Pte Ltd, 79 Ayer Rajah Crescent, Singapore 139955, Singapore; ^3^Institute of Liver Diseases, Shuguang Hospital, Shanghai University of Traditional Chinese Medicine, Shanghai, China; ^4^University of Utah Health Sciences Center, Salt Lake, UT, USA; ^5^Huntington Medical Research Institutes, Pasadena, CA, USA; ^6^Southern California Liver Centers, Riverside, CA, USA; ^7^Southern California Liver Centers, San Clemente, CA, USA; ^8^Stanford University, Veterans Affairs Palo Alto Health Care System, Palo Alto, CA, USA; ^9^Advanced Medical Research Center, Port Orange, FL, USA; ^10^Center for Drug Clinical Research, Shanghai University of Chinese Medicine, Pudong New District, Shanghai, China; ^11^Hepatic and Gastrointestinal Pathology, Pacific Rim Pathology Group, Sharp Memorial Hospital, San Diego, CA, USA; ^12^Department of Pathology of Shanghai Medical College of Fudan University, Shanghai, China; ^13^National Cancer Institute/National Institute of Health, Rockville/Bethesda, MD, USA; ^14^Advanced Liver Therapies, Baylor College of Medicine, Houston, TX, USA

## Abstract

**Background:**

Hepatitis C virus (HCV) is a common cause of progressive hepatic fibrosis, cirrhosis, and hepatocellular carcinoma worldwide. Despite the availability of effective direct-acting antivirals, patients often have significant hepatic fibrosis at the time of diagnosis due to delay in diagnosis and comorbidities which promote fibrogenesis. Thus, antifibrotic agents represent an attractive adjunctive therapy. Fuzheng Huayu (FZHY), a traditional Chinese medicine botanical formulation, has been used as an antifibrotic agent in chronic HBV infection. Our aim was to assess FZHY in patients with HCV infection and active viremia.

**Method:**

We randomized 118 patients with active viremia from 8 liver centers in the U.S. to receive oral FZHY (*n* = 59) or placebo (*n* = 59) for 48 weeks. Efficacy was assessed by histopathologic changes at the end of therapy. A subset of biopsies was further analyzed using qFibrosis to detect subtle changes in fibrosis in different zones of the hepatic lobules.

**Results:**

FZHY was well tolerated and safe. Patients with baseline Ishak fibrosis stages F3 and F4 had better response rates to FZHY than patients with baseline F0–F2 (*p*=0.03). qFibrosis zonal analysis showed significant improvement in fibrosis in all zones in patients with regression of the fibrosis stage.

**Conclusions:**

FZHY produced antifibrotic effects in patients with baseline Ishak F3 and F4 fibrosis stages. Reduction in fibrosis severity was zonal and correlated with the severity of inflammation. Based on its tolerability, safety, and efficacy, FZHY should be further investigated as a therapy in chronic liver diseases because of its dual anti-inflammatory and antiibrotic properties. *Lay Summary*. This is the first US-based, multicenter and placebo-controlled clinical trial that shows statistically significant reduction in fibrosis in patients with active HCV using an antifibrotic botanical formula. This has important implications as there is an immediate need for effective antifibrotic agents in treating many chronic diseases including NASH that lead to scarring of the liver. With artificial intelligence-based methodology, qFibrosis, we may provide a more reliable way to assess the FZHY as a therapy in chronic liver diseases because of its dual anti-inflammatory and antifibrotic properties.

## 1. Introduction

Hepatitis C virus (HCV) infection is one of the major health problems worldwide with an estimated 185 million or more individuals being infected with HCV around the globe [1–3]. Untreated chronic hepatitis C (CHC) can result in progressive necroinflammation, leading to the development of hepatic fibrosis which can progress to cirrhosis and complications of portal hypertension, hepatocellular carcinoma (HCC), and liver failure [2, 4, 5]. The prevalence of HCV infection is expected to decline with worldwide efforts to eliminate HCV infections via the use of interferon (IFN)-free, direct-acting antiviral agents (DAAs). Despite these efforts, the incidences of the morbid HCV outcomes, including hepatic fibrosis, cirrhosis, HCC, and liver failure are projected to rise during the next 20 years [2, 6–8].

Antifibrotic agents are currently in development as adjunctive therapies to retard or stop the progression of hepatic fibrosis and its complications after curative therapy for HCV [9, 10]. Fuzheng Huayu (FZHY), a traditional Chinese medicine (TCM), combining six ingredients in a botanical formula, has been shown to be effective in the treatment of hepatic fibrosis and cirrhosis caused by several underlying liver diseases, especially chronic hepatitis B virus (HBV) infection [11–14]. Multiple studies in experimental animals and isolated liver cells have proven that FZHY can reduce hepatic fibrosis. Studies on human subjects with chronic hepatitis B (CHB) and fibrosis in China have demonstrated efficacy in preventing the progression of chronic liver injury and hepatic fibrosis [12, 13, 15, 16].

In animal models, FZHY substantially inhibited the development of hepatic fibrosis induced by either CCL4 or bile duct ligation (BDL) [17]. FZHY downregulated mRNA transcripts for *α*-SMA, collagen- *α*1, tissue inhibitor of the matrix metalloproteinase-1 (TIMP-1), TGF-*β*1 and its receptor TGF-*β*R1, and platelet-derived growth factor-*β* (PDGF-*β*) [18]. These combined effects inhibit the transformation of stellate cells to fibrogenic myofibroblasts and their proliferation. FZHY also decreased the recruitment of F4/80+ inflammatory macrophages to the injured liver, and downregulated mRNA expression of monocyte chemoattractant protein-1 (MCP-1) and macrophage inflammatory protein-1 (MIP-1) [19]. In addition, FZHY effectively blocked the production of reactive oxygen species (ROS) in animal models of liver injury [20].

In humans, FZHY has been used in patients with CHB-related fibrosis since the late 1990s. Previous clinical studies demonstrated its ability to decrease liver inflammation and caused regression of the fibrosis stages. These effects were attributed to multiple mechanisms of action of FZHY that affect multiple fibrosis pathways [12]. Furthermore, FZHY has been shown to enhance degradation of hepatic fibrosis, improve serum aminotransferase and serum albumin levels, and protect hepatocytes from injury and death [12, 13]. Clinical studies have also shown that FZHY was well tolerated and had a good safety profile.

The aim of the current study was to assess the safety, tolerability, and efficacy of FZHY in the treatment of a group of U.S. adults with hepatic fibrosis due to chronic hepatitis C (CHC) and viremia who had failed prior anti-HCV IFN-based therapy. Histopathology was assessed using the Ishak scoring system to quantify hepatic inflammation and the fibrosis stage. In addition, fibrosis and inflammation were assessed using the sensitive qFibrosis system [21–23] to enhance the detection of meaningful changes in fibrosis during the study period of only 48 weeks [24].

## 2. Materials and Methods

### 2.1. Patients and Study Design

This was a phase 2b, randomized, placebo-controlled, double-blinded, multicenter study (NCT00854087). Of a total of 249 HCV-infected patients screened in 8 US. centers, 118 were randomized to receive either FZHY (*n* = 59) or placebo (*n* = 59). The key inclusion criteria included the following: [1] active CHC based on documentation of a positive serum anti-HCV antibody test and HCV RNA ≥50 IU/mL; [2] previous failure of IFN-based anti-HCV therapy or intolerance to or refusal of such therapy; [3] liver histology showing Ishak fibrosis score of 2, 3, or 4 in a liver biopsy performed within one year of screening. The principal exclusion criteria included the following: [1] history of human immunodeficiency virus (HIV) infection; [2] CHB; [3] uncontrolled diabetes or thyroid disease; [4] history of hepatic decompensation; [5] another comorbid chronic liver disease; [6] IFN-based antiviral treatment for HCV infection within six months of screening; or [7] treatment with investigational antiviral agents within 28 days prior to screening. A full list of inclusion and exclusion criteria is provided in the supplementary section.

Study subjects were randomized using an interactive web-based response system (IWRS) to receive either 48 weeks of oral treatment with FZHY or the matching placebo. All subjects were observed for an additional 12 weeks of treatment-free follow-up. All participants provided written informed consent before enrolment. This study was in accord with the Declaration of Helsinki and was consistent with the International Conference on Harmonization (ICH) Good Clinical Practice (GCP) and applicable regulatory requirements. The institutional review board (IRB) of each study site approved the study.

### 2.2. Treatment and Follow-Up

All randomized patients received treatment with FZHY 800 mg tablets at a dose of two tablets, three times a day (4.8 grams daily), or a matching placebo for 48 weeks. Further information on the formulation and quality of control of FZHY tablets and placebo are provided in the supplementary section. After completion of the 48-week treatment phase, patients entered the 12 weeks of treatment-free follow-up phase. Safety, tolerability, and efficacy assessments were conducted throughout the study. Histopathological evaluation was performed independently by three experienced pathologists in a blinded manner. A data safety and monitoring committee monitored safety throughout the study.

### 2.3. Histological Assessment

The primary efficacy endpoint was the change in the Ishak hepatic fibrosis stage from the baseline biopsy to the posttreatment biopsy [25]. “Fibrosis improved” was defined as an improvement in fibrosis by at least one stage, from baseline. “Fibrosis did not change” was defined as having the same Ishak score pretreatment and posttreatment. “Fibrosis worsened” was defined as an increase of at least one stage from baseline. Inflammation changes between baseline and posttreatment biopsies were also assessed in a similar manner.

Histopathological examinations at the start of the study were conducted by local pathologists to determine eligibility for entry into the study (Ishak stage 2, 3, and 4). At the end of the study, histopathologic examinations of baseline and posttreatment biopsies were performed by three hepatopathologists, who determined the Ishak fibrosis score and inflammation grade in each biopsy while blinded to whether the patients received FZHY or placebo and whether the biopsy was baseline or posttreatment. All three pathologists resolved differences in their grading and staging through consensus. After finalizing the grade and stage, the data was used to conduct paired analyses of the inflammation grade and the fibrosis stage using the baseline and posttreatment biopsies from each subject. The efficacy endpoints included [1] proportion of subjects regressing, ≥1 point on the Ishak fibrosis score at week 48, [2] change from the baseline fibrosis stage, and [3] ranked histological assessment of the paired biopsies for each patient (baseline vs. posttherapy at week 48).

### 2.4. qFibrosis Assessment

Unstained tissues of 74 paired liver biopsy samples were imaged using the Genesis®200 system of second harmonic generation (SHG) microscopy to visualize collagen, and two-photon excited fluorescence (TPEF) microscopy was used to visualize other cell structures. Images were acquired at 20x magnification with 512 × 512 pixels resolution, and each image tile had a dimension of 200 × 200 *μ*m. Multiple adjacent image tiles were captured to encompass the entire tissue area on each slide.

**Figure 1:**
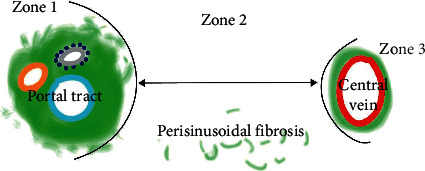
Schematic illustration of zones 1, 2, and 3 as applied in zonal analysis.

qFibrosis analyses were conducted using the methodology [23] previously reported in multiple studies [21–24, 26]. Since the fibrosis may vary within different zones of the hepatic lobule based on the etiology of the liver disease, qFibrosis staging was performed in all three hepatic zones as shown in Figure 1. Specifically, fibrosis was automatically detected and quantified in zone 1 (portal-periportal), zone 2 (sinusoidal), and zone 3 (central venous) [27–29]. A total of 100 morphological collagen parameters were quantified from each slide, including the percentages of different collagen patterns (all collagen, aggregated collagen, and distributed collagen) and collagen string features (such as the number of strings, numbers of short/long/thick/thin strings, and numbers of short/long/thick/thin aggregated/distributed strings).

**Figure 2:**
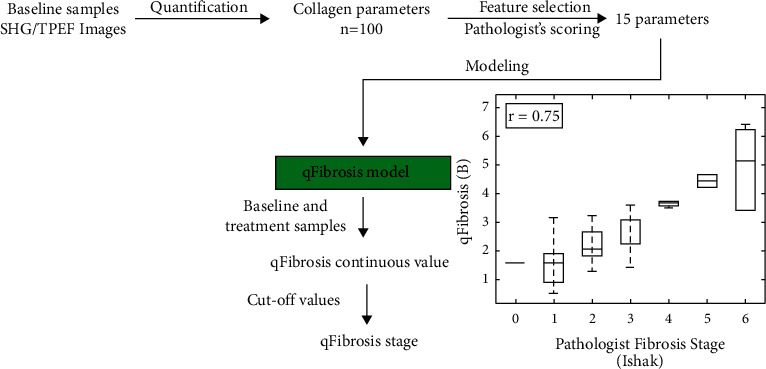
Workflow of the qFibrosis model used to assess pathologists' quantification consistency.

All baseline biopsies were used to train the qFibrosis model. A sequential feature selection method was used to select a subset of quantified features [30]. All baseline samples were used to find the most significant collagen features (fifteen features) related with the Ishak fibrosis staging. The process is summarized in the flow chart shown in Figure 2.

### 2.5. Assessment of Safety and Tolerability

Safety and tolerability of FZHY were assessed in study subjects from baseline through Week 60. Safety was evaluated through the changes in vital signs, physical examinations, adverse events (AEs), concomitant medications, and laboratory tests. AEs during the study were documented with dates of onset and resolution, severity, and outcome.

### 2.6. Sample Size Calculation

Based on previous studies of FZHY in patients with CHB (12), a total of 50 subjects in each arm was needed to detect a moderate effect size. A total of 100 subjects would achieve an 85% power with an alpha level of 0.05. We assumed a 25% difference in the proportion of subjects having a 1-point improvement according to Ishak fibrosis between the two groups as well as a 10% drop-out rate.

### 2.7. Randomization and Blinding

Eligible patients were randomized by an independent statistician in a 1 : 1 ratio to either receive treatment with FZHY or placebo. Randomization was centralized and a randomized schedule was generated prior to the initiation of the study. We used the identical placebo tablets to blind the investigator, and study staff and patients throughout the study. However, the pharmacist at each site was unblinded for safety reasons.

### 2.8. Statistical Analysis

Ratios for progressive, no change, and regressive fibrosis after treatment were calculated based on the change of the consensus fibrosis stage between baseline and end-of-treatment biopsies. The chi-squared test was used to compare regressive ratios between different groups. The qFibrosis model was trained on 74 baseline specimens and validated using the leave-one-out method [31]. Spearman's correlation was calculated to evaluate the models. Zonal analysis calculated the average change of collagen morphological parameters in zone 1, zone 2, and zone 3. The differences between FZHY and placebo treatments were assessed using the two-sample *t*-test method. The significance level was set at 0.05.

## 3. Results

### 3.1. Clinical Trial Population

**Figure 3:**
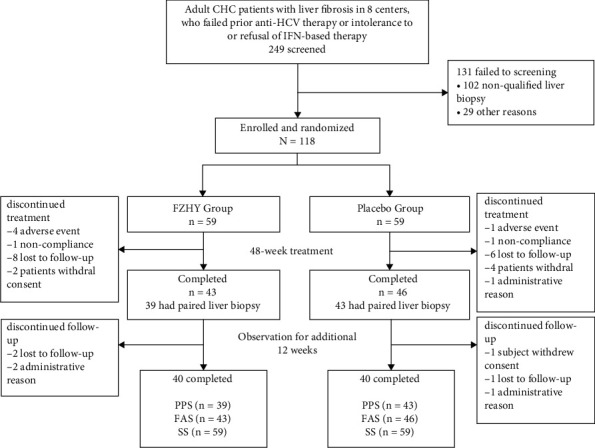
Flowchart of the patients enrolled in the FZHY clinical trial. The full-analysis set (FAS) 43/46 for patients who meet the inclusion criteria. The per-protocol set (PPS) 82 (39/43 with paired slides pretreatment and posttreatment in the FZHY and placebo groups) for primary endpoint assessment. The safety set (SS) 59/59 for all randomized patients for safety assessment.

Of the 249 patients who were screened, 118 were ultimately randomized: 59 patients were allocated to the FZHY treatment group and 59 to the placebo group. Among the 118 patients randomized, 82 patients (39 in the treatment group and 43 in the placebo group) had consensus Ishak fibrosis scores for both their baseline and posttreatment biopsies (Figure 3).

The demographics of the randomized patients are shown in Table 1, with no statistically significant difference between the FZHY and placebo groups.

### 3.2. Histological Assessment

**Figure 4:**
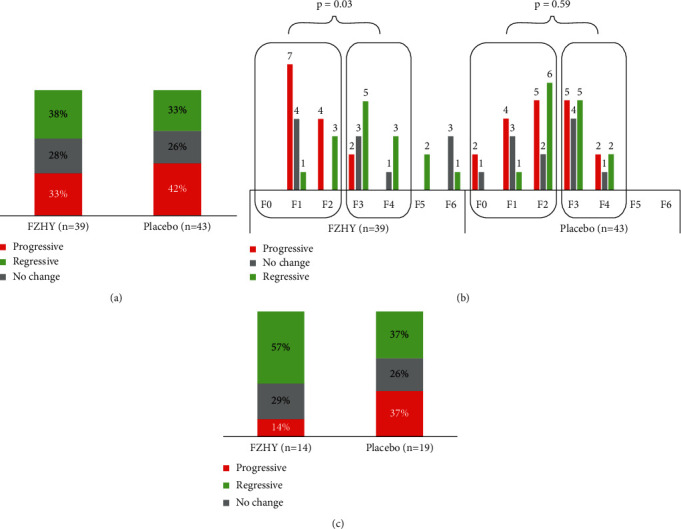
Fibrosis response (progressive/regressive/no change) obtained from the consensus reading of 3 pathologists. (a) Overall fibrosis response for all patients (*n* = 82) with the FZHY-treated group showing a slight improvement over the placebo group, in which the progressive group (*P*) is defined as fibrosis stage increased by ≥1 stage after treatment, no change group (*N*) as no change in fibrosis stages, and regressive group (*R*) as fibrosis stage decreased by ≥1 stage; (b) a closer look into the fibrosis response by categorizing according to the patients' baseline fibrosis staging. For patients in the FZHY group, those with baseline Ishak fibrosis F3 and F4 were observed to be more responsive toward treatment as compared to patients with baseline F0-2 (*p*=0.03). The chi-squared test was performed by comparing the number of responsive patients (*R*) with nonresponsive patients (*P*) and (*N*) between F0-2 and F3-4 groups; (c) focusing on patients with baseline fibrosis F3 and F4, the FZHY-treated group showed a more significant improvement over the placebo group based on the consensus reading.

The paired biopsy samples (PBS) from 82 patients were assessed independently by three pathologists from the U.S. and China, who were blinded as to whether the biopsies were baseline or posttreatment. To overcome interobserver variability among the three pathologists, a consensus reading was required to resolve any differences in the grading and staging. To achieve consensus, the three pathologists reassessed the biopsies in real time and reached a consensus for the grading and staging. The results are summarized in Table 1 of the supplementary section. The consensus fibrosis response at the end of treatment is shown in Figure 4 for both the FZHY and placebo groups.

The results indicated that patients treated with FZHY had a superior fibrosis response than those receiving placebo. The terms used to describe the patients' fibrosis responses i.e., progressive (*P*), no change (*N*), and regressive (*R*) [*P*/*N*/*R*] were defined as Ishak fibrosis stages increasing by ≥1 stage, Ishak fibrosis stages remaining the same, and Ishak fibrosis stages decreasing by ≥1 stage between baseline and week 48, respectively.

The fibrosis responses of patients receiving FZHY and placebo were also categorically analyzed based on their baseline fibrosis stages, as shown in Figure 4(b). The treatment group with baseline Ishak fibrosis stages F3 and F4 had a better response than patients with baseline Ishak fibrosis stages F0–F2 (*p*=0.03). Out of 14 patients with baseline F3 or F4, eight (57%) showed fibrosis regression and 2 (14%) showed fibrosis progression. In contrast, 19 patients with baseline F0, F1, or F2; four (21%) showed fibrosis regression; 11 (58%) showed fibrosis progression. In the placebo group, 37% of patients with baseline F3 or F4 showed fibrosis regression, compared to 29% of patients with baseline F0, F1, or F2. Fibrosis progressed in 37% of patients with baseline F3 or F4 and in 46% of patients with baseline F0, F1, or F2 (*p*=*NS*).

Subsequently, the P/N/R analysis was carried out on the subset of patients with baseline F3 and F4 fibrosis to evaluate their responses (Figure 4(c)). FZHY-treated patients with F3 or F4 showed a numerical improvement in fibrosis response compared with patients receiving placebo, but the improvement was not statistically significant (*p*=0.24).

### 3.3. qFibrosis Consistency Measurement

As mentioned earlier, independent fibrosis assessments by three pathologists showed significant interobserver variability with a measured kappa value of 0.376. This kappa value is considerably lower than that typically reported in studies of fibrosis (0.67–0.82) [32]. Since systematic discrepancies in grading and staging liver biopsies are common among expert pathologists, recent clinical trials have used a single pathologist to minimize interobserver variability in trial results [33, 34]

**Figure 5:**
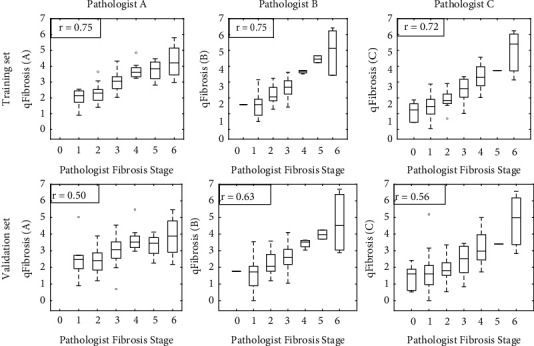
Box-whisker plots of qFibrosis component distribution relative to the Ishak scoring in the training and validation datasets. The Ishak fibrosis staging from pathologists A, B, and C is based on patients' baseline samples. For each stage, the maximum and minimum values are indicated by horizontal lines at the bottom and top of each stage, the white box in the middle represents data points in the 25% to 75% interquartile range, and the line through the middle of the central white box represents the median value. Note: *r* value was calculated according to the Spearman method.

As shown in Figure 5, qFibrosis analysis was employed to evaluate the consistency of the fibrosis assessments using the Ishak scores from the three pathologists with the respective R-values for each data set corresponding to the three pathologists. *R*-values were used to identify the pathologist with the highest *R* value, indicating who was comparatively the most consistent among the three. Pathologist B demonstrated an overall higher *R*-value for both the training and validation sets. Based on this finding, further analyses used only the grading and staging of pathologist B. It should be noted that for the qFibrosis assessment, only 74 patients with paired unstained slides were included for the analysis because there were no spare unstained slides available for eight patients.

**Figure 6:**
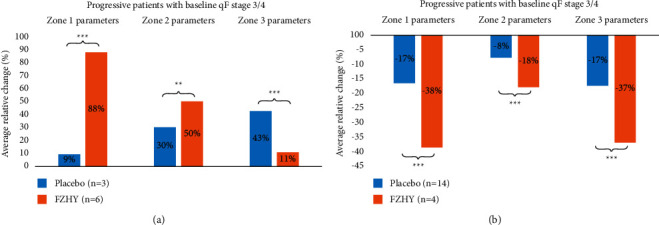
Zonal analysis was conducted on the progressive and regressive patient subsets with baseline qFibrosis stages of F3 and F4 with statistical significance shown across zones 1, 2, and 3. FZHY-treated patients in the regressive subset showed significant fibrosis reduction as compared to the placebo group consistently across all zones. FZHY-treated patients in the progressive subset showed considerable fibrosis increment over the placebo group, particularly in zone 1.

Having established from the consensus results shown in Figure 4(b) that baseline F3 and F4 patients were responsive to FZHY treatment compared to other fibrosis stages; we conducted an in-depth analysis of the regressive and progressive patient subsets. Specifically, qFibrosis zonal analysis was conducted in patients who had been staged as F3 and F4 fibrosis by pathologist B (Figure 6).

Data in Figure 4(c) shows that in patients with F3 or F4 fibrosis at baseline, FZHY treatment resulted in more fibrosis regression than that observed in the placebo group. This was corroborated by qFibrosis zonal analysis as shown in Figure 6(b). Patients with regressive fibrosis showed reduction across all three hepatic zones in both the placebo and treatment groups. However, patients treated with FZHY showed significantly greater fibrosis reduction when compared to the patients receiving placebo.

In the subset of patients with progressive fibrosis, significant differences were observed between patients treated with FZHY vs. placebo in zones 1 and 3. However, placebo-treated patients showed a greater degree of progressive fibrosis in zone 3 fibrosis compared with FZHY-treated patients. Among patients with progressive fibrosis, FZHY-treated patients had a greater increase in zone 1 fibrosis than the placebo-treated patients. This zone 1 difference may be explained, in part, by a higher level of baseline fibrosis in patients randomized to receive FZHY vs. placebo.

### 3.4. Correlation between Fibrosis and Inflammation in Zone 1

**Figure 7:**
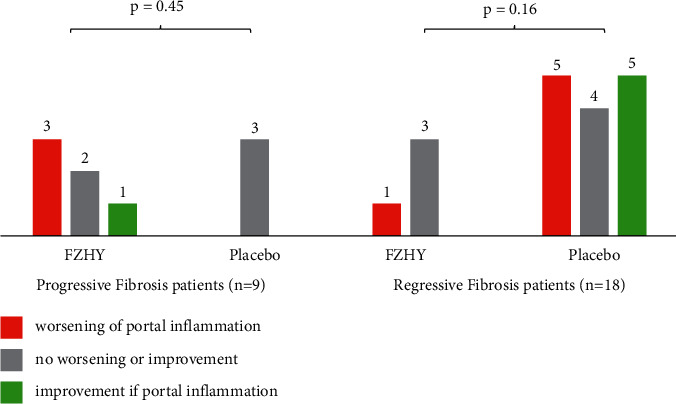
Portal inflammation analysis on the progressive and regressive patient subsets with baseline qFibrosis stages of F3 and F4. No statistical significance was reported between FZHY-treated and placebo groups due to the small sample size.

In the initial assessment of the consensus scores for the grade of inflammation, no statistically significant differences were noted between patients treated with FZHY or placebo (refer to the supplementary section, Tables 4.1 and 4.2). After identifying differences in qFibrosis zonal fibrosis responses, the inflammation grades were reassessed in patients with baseline F3 or F4 fibrosis who had shown progressive or regressive qFibrosis (Figure 7).

The grade of inflammation in zone 1 was higher in patients with progressive fibrosis who had been treated with FZHY than in patients receiving placebo or patients with regressive fibrosis. This affirmed the direct correlation between inflammation and fibrosis. However, there was no statistically significant difference between the placebo and treatment groups, which may reflect the small number of patients analyzed for portal inflammation.

### 3.5. Safety and Tolerability Assessment of FZHY in the Treatment of HCV-Associated Hepatic Fibrosis

**Figure 8:**
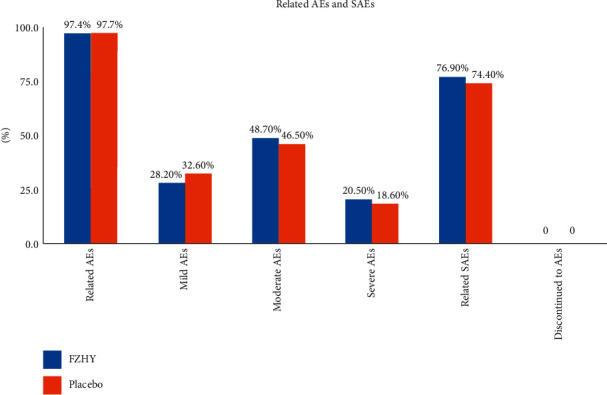
Antifibrotic FZHY treatment-related AEs and SAEs of all grades. No significant SAEs were reported that were related to the study drug.

Safety and tolerability were assessed in 98 patients who were >80% compliant with the treatment regimens (Figure 8 and supplementary Table 5). A total of 17 serious AEs occurred, but none were adjudicated to have been related to FZHY. There was no significant difference in adherence, AEs, serious AEs, or any of the laboratory parameters between the FZHY and placebo groups. AEs associated with FZHY or placebo were mild to moderate in intensity in 99.6% of patients.

Among the AEs, gastrointestinal complaints were the most common even though their frequency was not significantly different between patients receiving FZHY (66.1%) or placebo (50.8%). AEs involving general health, infections, and skin disease were more frequent in the FZHY group (45.8%, 40.7%, and 23.7%, respectively) compared with those in the placebo group (35.6%, 33.9%, and 22%, respectively). In contrast (Table 6 in the supplementary section), the frequencies of AEs involving the respiratory and nervous system, as well as procedural complications were increased in the placebo group vs. the FZHY group (40.7% vs.33.9%, 35.6% vs. 33.9%, and 20.3% vs. 16.9%, respectively). Alanine aminotransferase (ALT) and aspartate aminotransferase (AST) levels decreased during treatment with FZHY and increased after cessation. The HCV viral load did not change in FZHY-treated patients. Additional details are present in supplementary Table 7.

## 4. Discussion

This is the first clinical trial in the U.S. to investigate the tolerability, safety, and efficacy of FZHY, a botanical formula with antifibrotic activity that has been well established in China as a traditional Chinese medicine (TCM). The formula includes Radix Salviae miltiorrhizae and Salvianolic acid B, which have proven antifibrotic mechanisms of action. The FZHY used in this study was produced under strict good manufacturing practices to guarantee a uniform quality. The randomized, placebo-controlled, double-blind study addressed the unmet need to retard or reverse hepatic fibrosis in patients who failed or could not tolerate IFN-based antiviral therapy. The study confirmed the tolerability and safety of FZHY observed in China in U.S. patients with viremic CHC. Although FZHY was only given for 48 weeks, its antifibrotic effects were observed by comparing liver biopsies at baseline with those obtained at the end of the treatment. Fibrosis was assessed using both the established Ishak fibrosis scoring system and the newer qFibrosis method which can detect and quantify subtle changes in fibrosis in all three zones of the hepatic lobules.

CHC patients who enrolled in this study had failed to achieve a sustained virologic response (SVR) using IFN-based therapy. Thus, these persistently viremic patients were at risk of fibrosis progression. The results showed that the anti-fibrotic effects of FZHY were prominent in patients with baseline Ishak F3 and F4 fibrosis, despite a duration of therapy of only 48 weeks. Among patients with regression in fibrosis on their posttreatment liver biopsies, evidence of regression was observed in all three zones of the hepatic lobule. In the subset of patients who showed progression of fibrosis, the severity was more prominent in zone 1 than in either zone 2 and 3.

The study findings also confirmed that this subset of patients had prominent inflammation in their portal tracts, likely indicative of the immune response induced by HCV replication. These results in patients with viremic CHC are in contrast to the significant improvement in fibrosis severity observed after FZHY treatment of patients with CHB and suppression of HBV replication using nucleoside/tide analogues vs. controls (52% vs. 23.3%, respectively) [12]. Our results raise the possibility that viremic CHC patients treated with FZHY may exhibit regressive fibrosis in some hepatic zones and progressive fibrosis in other zones in the same biopsy sample.

In this study, progressive and regressive features of fibrosis coexisted within the same biopsy slides. The results of the qFibrosis zonal analysis support the view that fibrosis in the hepatic lobule is a dynamic process in which the degree of fibrosis may vary in different zones at the same time. This poses a challenge to the current method of histopathological assessments of liver biopsies. This was also corroborated by the poor kappa value among the three expert pathologists whose differences in grading and staging could only be resolved by reaching a realtime consensus reading while blinded with respect to the treatment, and whether the biopsy was obtained at baseline or posttreatment. The consensus readings revealed that the antifibrotic effects of FZHY worked best for patients with baseline Ishak F3 and F4. The relative change in fibrosis within the F3/F4 patient cohort was zonal and most prominent in zone 1 in the presence of severe inflammatory infiltrates. Since all patients in the study had viremic CHC after failing IFN-based therapy, severe portal inflammation was present in both the FZHY and placebo groups.

The limitation of this study included the involvement of more than one pathologist from different countries, which required consensus readings for most aspects of inflammation and fibrosis severity, and underscored the importance of automation in the assessment of histopathology. Similar to other studies, the variability in the size of the liver tissue on the biopsy slides could play a role in the histologic interpretation done by each pathologist. The approach of requiring consensus reading minimized that limitation.

Another limitation of this study is its small sample size; a larger study with a longer treatment duration and the inclusion of more F3 and F4 subjects would be needed to fully assess the efficacy of FZHY on liver fibrosis.

## 5. Conclusions

This phase 2 clinical trial showed that FZHY was well tolerated and safe in patients with viremic CHC and fibrosis stages 2, 3, and 4. Comparative analyses of fibrosis in baseline and posttreatment biopsies using Ishak staging and qFibrosis methods indicated that FZHY had antifibrotic effects in patients with CHC. The antifibrotic effects of FZHY were most prominent in CHC patients with baseline Ishak stages F3 and F4 fibrosis. The qFibrosis analyses confirmed the efficacy of FZHY by demonstrating fibrosis regression in all the three zones of the hepatic lobule, despite active viremia and persistent inflammation. Thus, FZHY should be studied further as an antifibrotic agent in patients with chronic fibrotic liver diseases.

## Figures and Tables

**Table 1:** Demographic and baseline characteristics of randomized patients.

	FZHY (*N* = 59)	Placebo (*N* = 59)	*p* value
Age (years)	55.4 ± 6.72	55.1 ± 8.6	0.849
Gender (% male)	57.6%	61.6%	0.708
Ethnicity			0.206
Caucasian	79.7%	78.0%	
African American	15.3%	11.9%	
Others	5.0%	10.1%	
HCV genotype			0.619
1	81.4%	88.1%	
2	6.8%	1.7%	
3	10.2%	8.5%	
Others	1.7%	1.7%	
History of IFN use			0.822
Yes	79.7%	78.0%	
No	20.3%	22.0%	

## Data Availability

The data used to support the findings of this study are included within the article, in the supplementary material file, as well as on clinicaltrials.gov NCT 00854087.

## Abbreviations

ALT: Alanine transaminase
AST: Aspartate transaminase
CHC: Chronic hepatitis C
DAAs: Direct-acting antiviral agents
FZHY: Fuzheng Huayu
GCP: Good clinical practice
HBV: Hepatitis B virus
HCC: Hepatocellular carcinoma
HCV: Hepatitis C virus
ICH: International conference on harmonization
IFN: Interferon
IRB: Institutional review board
LC: Liver cirrhosis
MCP-1: Monocyte chemoattractant protein-1
MIP-1: Macrophage inflammatory protein-1
PDGF-*β*: Platelets-derived growth factor-*β*
ROS: Reactive oxygen species
SHG: Second harmonic generation
TCM: Traditional Chinese medicine
TIMP-1: Tissue inhibitor of matrix metalloproteinase-1
TPEF: Two-photon excitation fluorescence.

## Supplementary Materials

Supplementary Materials Table 1: Fuzheng Huayu (FZHY) formulation (g/daily dose). Figure 1. General manufacturing protocol for FZHY tablet. Table 2: component markers standard for FZHY. Figure 2. The chromatographic profile of FZHY extracts (stationary phase: CNW Athena C18-WP (4.6 mm × 150 mm, 3 *μ*m), flow rate: 1 mL/min)). Peak No: (1) danshensu; (2) rotocatechuic aldehyde; (3) rosmarinic acid; (4) salvianolic acid B; (5) schizandrol A; (6) schizandrol B; (7) schizandrin A; (8) uridine (9) guanosine; (10) adenosine. Table 3: Ishak fibrosis staging based on consensus reading by 3 pathologists. Table 4.1: paired assessment of liver Ishak HAI (histological per-protocol population). Table 4.2: paired assessment of liver Ishak portal inflammation (qFibrosis analysis population). Table 5: summary of treatment emergent adverse events and serious adverse events (histological per-protocol population). Table 6: summary of treatment emergent adverse events by system organ class (histological per-protocol population). Table 7: summary of treatment emergent adverse events by severity (histological per-protocol population). Figure 3. The change of ALT and AST levels between FZHY (*n* = 39) and placebo (*n* = 43) groups during treatment and follow-up. ALT and AST levels decreased in the FZHY group during treatment, but increased after the end of treatment. ALT: alanine transaminase; AST: aspartate transaminase.
