# Supplementary material for: Efficacy and Safety of a Botanical Formula Fuzheng Huayu for Hepatic Fibrosis in Patients with CHC: Results of a Phase 2 Clinical Trial
Source: Evid Based Complement Alternat Med. 2022 Jul 15;2022:4494099. doi: 10.1155/2022/4494099 (PMC9307334; doi:10.1155/2022/4494099)
Supplement: Supplementary Materials — Table 1: Fuzheng Huayu (FZHY) formulation (g/daily dose). Figure 1. General manufacturing protocol for FZHY tablet. Table 2: component markers standard for FZHY. Figure 2. The chromatographic profile of FZHY extracts (stationary phase: CNW Athena C18-WP (4.6 mm × 150 mm, 3 μm), flow rate: 1 mL/min)). Peak No: (1) danshensu; (2) rotocatechuic aldehyde; (3) rosmarinic acid; (4) salvianolic acid B; (5) schizandrol A; (6) schizandrol B; (7) schizandrin A; (8) uridine (9) guanosine; (10) adenosine. Table 3: Ishak fibrosis staging based on consensus reading by 3 pathologists. Table 4.1: paired assessment of liver Ishak HAI (histological per-protocol population). Table 4.2: paired assessment of liver Ishak portal inflammation (qFibrosis analysis population). Table 5: summary of treatment emergent adverse events and serious adverse events (histological per-protocol population). Table 6: summary of treatment emergent adverse events by system organ class (histological per-protocol population). Table 7: summary of treatment emergent adverse events by severity (histological per-protocol population). Figure 3. The change of ALT and AST levels between FZHY (n = 39) and placebo (n = 43) groups during treatment and follow-up. ALT and AST levels decreased in the FZHY group during treatment, but increased after the end of treatment. ALT: alanine transaminase; AST: aspartate transaminase. [file 4494099.f1.pdf]

Efficacy and safety of a botanical formula Fuzheng Huayu for hepatic fibrosis in patients with Chronic Hepatitis C: A phase II randomized, double-blinded, multicenter trial

Supplementary Section

Methods

Inclusion Criteria

1. Signed, written, informed consent must be available from the subject or legal representative before any study-specific procedures are performed.
2. Male or female 18-70 years of age.
3. Chronic hepatitis C infection based on documented history of a positive serum anti-HCV antibody test and/or detectable levels of HCV RNA ⩾ 50 IU/mL.
4. Failure to achieve sustained Virologic response (SVR) with previous Interferon based therapy or subjects who refuse Interferon based therapy or are intolerant to Interferon.
5. Liver biopsy within 1 year of the Screening visit and at the time the biopsy was performed the subject has not received Interferon based treatment within 6 months, if applicable. The liver biopsy must document chronic liver disease consistent with chronic hepatitis C and an Ishak fibrosis score of 2, 3, or 4 as determined by a local pathologist.
6. All subjects enrolling in the study and all fertile or potentially fertile sexual partners of subjects must be using two reliable forms of effective contraception during the study unless a study participant/partner is surgically sterile or postmenopausal.

Exclusion Criteria

1. Subjects with any history of decompensated liver disease, including but not restricted to portal hypertension as manifested by gastroesophageal varices, variceal bleeding, ascites, or encephalopathy or a hepatic mass lesion suspicious for hepatocellular carcinoma (HCC).
2. Liver histology consistent with any other co-existing cause of chronic liver disease (apart from fatty liver).
3. Any of the following laboratory abnormalities at Screening:
   - Hemoglobin <12 g/dL for females, and <13 g/dL for males.
   - White blood cell count ⩽3,000/mm3.
   - Absolute neutrophil count ⩽2,000/mm3.
   - Platelet count ⩽75,000/ mm3.

- – Total bilirubin ≥1.4 mg/dL (with the exception of subjects with Gilbert's syndrome).
- – Albumin <3.2g/dL.
- – Hemoglobin A1C level ≥8.5% in diabetics.
- – Serum creatinine above upper limit of normal (ULN).
- – Serum ALT >5 × ULN.
- – Alpha-fetoprotein >200ng/mL.
- – Prothrombin time >15 seconds or International normalized ratio (INR) >1.3.

4. Subjects who have been treated for HCV infection within 6 months before Screening.
5. Subjects who have been on any experimental protocol or therapy within 28 days before Screening.
6. Known HIV infection.
7. Chronic hepatitis B infection as defined by a positive HBs Ag.
8. Uncontrolled diabetes.
9. Unstable or uncontrolled thyroid disease (subjects requiring medication to control their thyroid disease are eligible if all other inclusion/exclusion criteria are met).
10. Presence or history of non-HCV chronic liver disease, including autoimmune hepatitis, alpha-1-antitrypsin deficiency, hemochromatosis, Wilson's disease, drug- or toxin-induced liver disease, alcohol-related liver disease, primary biliary cirrhosis and sclerosing cholangitis. Subjects with fatty livers in addition to HCV will be included in the study.
11. History of unstable or deteriorating cardiovascular or cerebrovascular disease within 6 months before Screening and/or QTcF ≥ 450 ms at the Screening visit.
12. Uncontrolled seizures disorder.
13. History of hemoglobinopathies, (e.g., thalassemia, sickle cell anemia, spherocytosis) or other cause of hemolytic anemia, including autoimmune causes.
14. History of malignant cancer within the last 5 years with the exception of localized basal or squamous cell carcinoma.
15. A disease known to cause significant alteration in immunologic function including hematologic malignancy, sarcoidosis or autoimmune disorder (e.g., rheumatoid arthritis, systemic lupus erythematosis, leukemia, lymphoma, autoimmune thyroid

disease, scleroderma, unstable psoriasis, inflammatory bowel disease, and multiple sclerosis).

16. History of major organ transplantation (i.e., liver, kidney, lung, or heart) with an existing function graft, including bone marrow transplant or stem cell transplant.
17. Concurrent therapy with immunosuppressive drugs or cytotoxic agents such as cyclosporine, azathioprine, chronic systemic corticosteroids, or chemotherapeutic agent(s) (e.g., cyclophosphamide, methotrexate, or cancer chemotherapy) or radiation therapy.
18. Alcohol and/or drug abuse within the past year.
19. Pregnant or lactating women or women who plan to become pregnant during the study.
20. Subjects with known sensitivity to Fuzheng Huayu or any of its components.
21. Subjects, who in the opinion of the Investigator, are not suitable candidates for enrollment or who would not comply with the requirements of the study.
22. Body Mass Index > 36 at the Screening visit.

Formulation of Fuzheng Huayu Tablet

All the raw materials for FZHY comes from the medicinal material planting and harvesting bases according to Good Agricultural And Collection Practices (GACP) requirement in definite places, and identified for their origin, morphological, microscopic, physical, chemical characteristics, as well as the DNA barcode identification to guarantee the authenticity and quality of medicinal materials.

Table 1: Fuzheng Huayu (FZHY) formulation (g/ daily dose)

Chinese name	Plant sources	Medicinal parts	Preparation amount (g)
**Danshen**	*Salvia Miltiorrhizae Bge (Labiatae)*	radix	8
**Chongcao**	artificial fermentation *cordyceps*	mycelia	4
**Taoren**	*Prunus persica* (L.) Batsch (Rosaceae)	fruit	2
**Jiaogulan**	*Gynostemma pentaphyllum* (Thunb)	whole herb	6
**Songhuafen**	*Pinus massoniana* Lamb (Pinaceae)	pollen	2
**Wuweizi**	*Schisandrae Chinensis* (Turcz.)Baill	fruit	2

Quality of Control of Fuzheng Huayu Tablet

Standard Manufacturing Process

For the preparation of FZHY extraction, 666g of Danshen, 500g of Jiaogulan, 334g of Chongcao, 166g of each, Taoren, Songhuafen and Wuweizi are weighed up. Danshen, Taoren and Jiaogulan are first mixed with appropriate amount of water to decoct twice, 2 hours for the first time and 1.5 hour for the second time. The decoctions are then combined and left to stand for 24h to allow the supernatant to concentrate until a relative density of about 1.20 (50–55℃). This is then cooled, and alcohol (95%) is added while slowly shaking up until the alcohol content reaches 70%. It is then cooled again, filtrated and concentrated until the relative density reaches 1.3–1.4 at 50–55℃. Drying takes place by decompression to obtain the dry extract. Then, Chongcao and Wuweizi are weighed up, reflux extraction with alcohol twice, 2 hours for the first time and 1.5 hour at the second time. The extract solution is combined, filtrated and concentrated until the relative density reaches 1.3–1.4 at 50–55℃. The alcohol is recovered and dried by decompression to get the dry extract. Similarly, Songhuafen is soaked in 50% alcohol at 40℃ twice, 4 hours for the first time and 2

hours for the second time. The extract solution is combined, filtrated and concentrated until the relative density reaches 1.3–1.4 at 50–55℃ The alcohol is recovered, dried and decompressed to obtain the dry extract. Those three dry extracts are mixed and dried as the FZHY extract powder to reserve, and the ratio of dry extract is 12.85%.

Then pharmaceutical excipients are added to the FZHY extract powder and the tablets are prepared directly by compression.

It can be compressed to 1000 tablets (0.4g each) or 500 tablets (0.8g each).

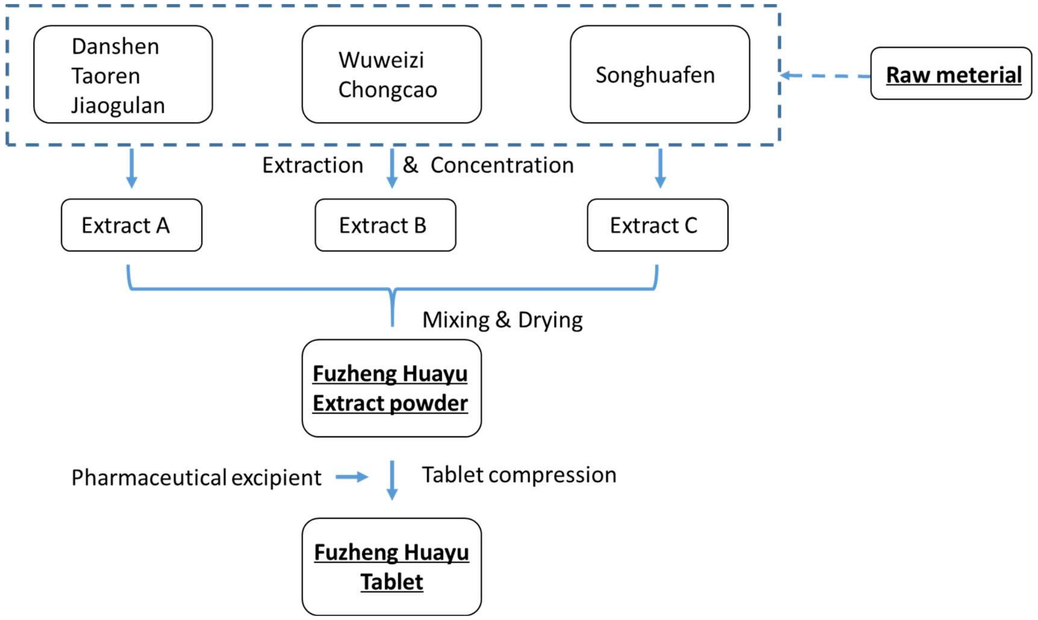

Figure 1. General manufacturing protocol for FZHY tablet.

Chemical Components Content of FZHY

Compounds (marker)	Quality criterion
Salvianolic acid B (from Danshen)	Should be no less than 15.6mg in 24g of FZHY raw materials (daily dose）
Sodium Danshensu (from Danshen)	Should be no less than 13.2mg in 24g of FZHY raw materials (daily dose）
Adenosine (from Chongcao)	Should be no less than 4.8mg in 24g of FZHY raw materials (daily dose)
Schisandrin B (from Wuweizi)	Should be no less than 2.28mg in 24g of FZHY raw materials (daily dose)

Table 2: Component markers standard for FZHY.

Multi-components assay (Fingerprinting)

To control the quality of the FZHY extracts, the fingerprint spectrum was established using high performance liquid chromatography (HPLC) method. Assay validation was performed according to the United States Food and Drug Administration (US FDA) bio-analytical method validation guideline (FDA, Center for Drug Evaluation & Research, 2001). The chromatographic profile of the extracts is shown in Figure 2. The contents of adenosine and danshensu were 2.5mg/g and 8.04mg/g in the extracts respectively, according to quality inspection report from the Shanghai Sundise Chinese Medicine Technology Development Co., Ltd (Shanghai, China). The ratio of dry extract is 12.85%.

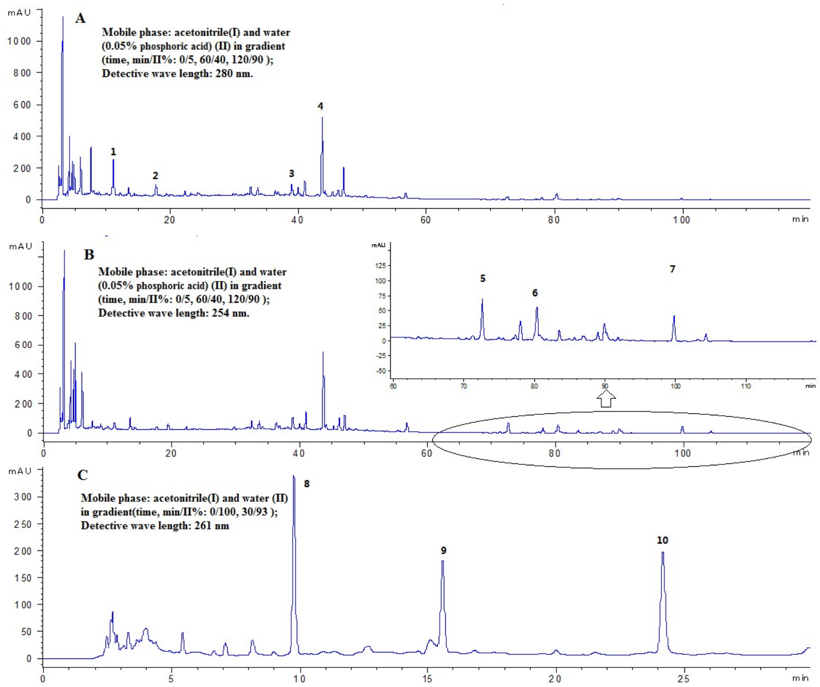

Figure 2. The chromatographic profile of FZHY extracts [Stationary phase: CNW Athena C18-WP (4.6mm×150mm, 3μm), flow rate: 1 mL/min). Peak No.: 1. danshensu; 2. protocatechuic aldehyde; 3. rosmarinic acid; 4. salvianolic acid B; 5. schizandrol A; 6. schizandrol B; 7. schizandrin A; 8. uridine 9. guanosine; 10. adenosine.

Results

Fibrosis responses based on consensus reading

3 pathologists re-assessed the biopsies to reach a consensus reading for all the samples, and the results are summarized in Table 3.

Ishak Fibrosis Staging	Number of patients	
	FZHY	Placebo
**Pre-Treatment Fibrosis Staging (p = 0.271)**		
2	13	21
3	17	12
4	9	10
**Pre-Treatment Consensus Fibrosis Staging (p = 0.043)**		
0	0	3
1	13	8
2	7	13
3	10	14
4	3	5
5	2	0
6	4	0
**Post-Treatment Consensus Fibrosis Staging (p = 0.504)**		
0	2	4
1	11	10
2	3	8
3	10	12
4	7	5
5	2	3
6	4	1

Table 3: Ishak fibrosis staging based on consensus reading by 3 pathologists.

Inflammation responses based on consensus scores

Table 4.1: Paired assessment of liver Ishak HAI (Histological per-protocol population)

Category	FZHY<br>N=39	Placebo<br>N=43	p-value
Better	15(38.5%)	17(39.5%)	0.921
No change	11(28.2%)	12(27.9%)	
Worse	13(33.3%)	14(32.6%)	

P-value is from a Wilcoxon rank sum test.

Table 4.2: Paired assessment of liver Ishak portal inflammation (qFibrosis analysis population)

Category	FZHY<br>N=35	Placebo<br>N=39	p-value
Better	7(20.0%)	8(20.5%)	0.84
No change	22(62.9%)	25(64.1%)	
Worse	6(17.1%)	6(15.4%)	

P-value is from a Wilcoxon rank sum test.

Safety and tolerability assessment of FZHY

The severity of the adverse events (AEs) was graded in accordance with the Common Terminology Criteria for Adverse Events (CTCAE) in which AEs are classified into different five grades with Grade 1 (mild), Grade 2 (moderate), Grade 3 (severe), Grade 4 (life threatening), and Grade 5 (death). Those AEs identified to be severe were reported using a tool for serious-adverse-event. The safety assessments were undertaken for each patient throughout the study, and laboratory tests were performed by a local central laboratory using standard procedures.

Table 5: Summary of treatment emergent adverse events and serious adverse events (Histological per-protocol population)

Category		FZHY<br>N= 39<br>N (%)	Placebo<br>N= 43<br>N (%)	p-value
**AE**				
	at least one AE	38(97.4%)	42(97.7%)	1.000
	no AE	1(2.6%)	1(2.3%)	
**SAE**				
	at least one SAE	7(17.9%)	7(16.3%)	1.000
	no SAE	32(82.1%)	36(83.7%)	
**Study drug related AE**				
	at least one study drug related AE	30(76.9%)	32(74.4%)	1.000
	no study drug related AE	9(23.1%)	11(25.6%)	
**Study drug related SAE**				
	at least one study drug related SAE	0(0.0%)	1(2.3%)	1.000
	no study drug related SAE	39(100.0%)	42(97.7%)	

P-values are from a Fisher's exact test.
A subject is counted only once at each row for multiple occurrences of an AE/SAE/ Study drug related AE.

Table 6: Summary of treatment emergent adverse events by system organ class (Histological per-protocol population)

Body system/preferred term	FZHY N= 39 No. of Subjects	No. of Cases	Placebo N= 43 No. of Subjects	No. of Cases
AE	38(97.4%)	330	42(97.7%)	334
Gastrointestinal disorders	30(76.9%)	67	24(55.8%)	66
General disorders and administration site conditions	21(53.8%)	34	20(46.5%)	26
Musculoskeletal and connective tissue disorders	18(46.2%)	44	23(53.5%)	46
Infections and infestations	18(46.2%)	28	18(41.9%)	31
Nervous system disorders	14(35.9%)	38	18(41.9%)	31
Psychiatric disorders	15(38.5%)	20	10(23.3%)	16
Respiratory, thoracic and mediastinal disorders/Immune system disorders	0(0.0%)	0	22(51.2%)	56
Skin and subcutaneous tissue disorders	12(30.8%)	15	10(23.3%)	13
Respiratory, thoracic and mediastinal disorders	19(48.7%)	35	0(0.0%)	0
Injury, poisoning and procedural complications	6(15.4%)	7	8(18.6%)	10
Surgical and medical procedures	6(15.4%)	9	6(14.0%)	6
Investigations	3(7.7%)	3	8(18.6%)	8
Metabolism and nutrition disorders	4(10.3%)	7	3(7.0%)	3
Renal and urinary disorders	6(15.4%)	7	1(2.3%)	1
Vascular disorders	2(5.1%)	2	5(11.6%)	7
Eye disorders	3(7.7%)	3	3(7.0%)	3
Cardiac disorders	2(5.1%)	2	3(7.0%)	3
Ear and labyrinth disorders	3(7.7%)	3	1(2.3%)	1
Immune system disorders	1(2.6%)	1	3(7.0%)	3
Neoplasms benign, malignant and unspecified (incl cysts and polyps)	3(7.7%)	3	0(0.0%)	0
Reproductive system and breast disorders	1(2.6%)	1	2(4.7%)	3
Endocrine disorders	0(0.0%)	0	1(2.3%)	1
Hepatobiliary disorders	1(2.6%)	1	0(0.0%)	0

'System/organ class' are defined in the Medical Dictionary for Regulatory Activities (MedDRA version 13.0).

Table 7: Summary of treatment emergent adverse events by severity (Histological per-protocol population)

AE/ Severity		FZHY<br>N= 39<br>No. of Subjects	No. of Cases	Placebo<br>N= 43<br>No. of Subjects	No. of Cases
**AE**		38(97.4)	330	42(97.7)	334
	Mild	11(28.2)	252	14(32.6)	243
	Moderate	19(48.7)	66	20(46.5)	75
	Severe	8(20.5)	12	8(18.6)	16
**Study drug related AE**		30(76.9)	120	32(74.4)	91
	Mild	14(35.9)	95	19(44.2)	67
	Moderate	16(41.0)	25	12(27.9)	23
	Severe	0(0.0)	0	1(2.3)	1
**Discontinuation due to AE**		0(0.0)	0	0(0.0)	0
	Mild	0(0.0)	0	0(0.0)	0
	Moderate	0(0.0)	0	0(0.0)	0
	Severe	0(0.0)	0	0(0.0)	0

A subject is counted only once with the most severity grade at No. of Subjects for multiple occurrences of an AE.

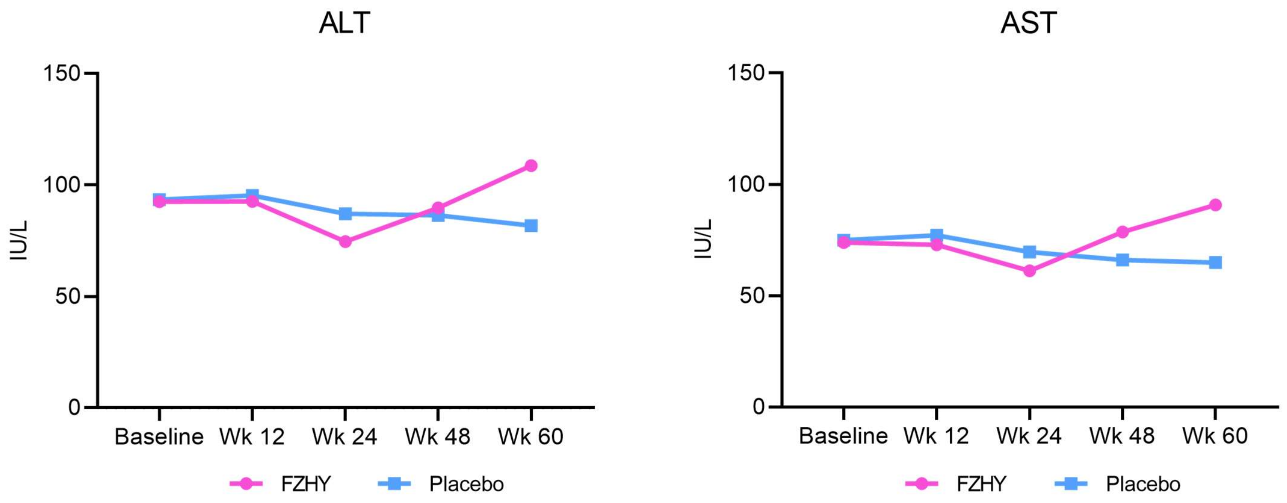

Figure 3. The change of ALT and AST levels between FZHY (n=39) and Placebo (n=43) group during treatment and follow-up. ALT and AST levels decreased in the FZHY group during treatment, but increased after the end of treatment. ALT,alanine transaminase; AST, aspartate transaminase.
